# Suppression of the Imprinted Gene *NNAT* and X-Chromosome Gene Activation in Isogenic Human iPS Cells

**DOI:** 10.1371/journal.pone.0023436

**Published:** 2011-10-12

**Authors:** Jonathan H. Teichroeb, Dean H. Betts, Homayoun Vaziri

**Affiliations:** 1 Ontario Cancer Institute, Department of Medical Biophysics, University of Toronto, Toronto, Ontario, Canada; 2 Department of Physiology and Pharmacology, Schulich School of Medicine & Dentistry, The University of Western Ontario, London, Ontario, Canada; Genome Institute of Singapore, Singapore

## Abstract

Genetic comparison between human embryonic stem cells and induced pluripotent stem cells has been hampered by genetic variation. To solve this problem, we have developed an isogenic system that allows direct comparison of induced pluripotent stem cells (hiPSCs) to their genetically matched human embryonic stem cells (hESCs). We show that hiPSCs have a highly similar transcriptome to hESCs. Global transcriptional profiling identified 102–154 genes (>2 fold) that showed a difference between isogenic hiPSCs and hESCs. A stringent analysis identified *NNAT* as a key imprinted gene that was dysregulated in hiPSCs. Furthermore, a disproportionate number of X-chromosome localized genes were over-expressed in female hiPSCs. Our results indicate that despite a remarkably close transcriptome to hESCs, isogenic hiPSCs have alterations in imprinting and regulation of X-chromosome genes.

## Introduction

Induction of pluripotency by reprogramming factors has been shown to result in creation of cells with properties similar to human embryonic stem cells [1], [2] (hESCs). Although human induced pluripotent stem cells (hiPSCs) are shown to mimic hESCs, several groups have shown that aberrations exist in hiPSCs when compared to hESCs [3], [4], [5]. These biological differences are exemplified by a large number of genes that show differential expression between hESCs and hiPSCs [1], [6], [7]. How closely the identities of hiPSCs derived in vitro by transcriptional reprogramming resemble those of embryo-derived hES cells has been an unmitigated question. Despite morphological and genetic similarities, global transcriptional comparison of hESCs and hiPSCs has revealed some significant differences. Several studies have identified as many as 1267–3947 genes with varying levels of deviation [1], [6], [7]. In all these cases genetic variation and dependence on the set of hiPSCs and hESCs used significantly confounds interpretation of the data [7]. We have developed a human isogenic system of induced pluripotency that we used in this study to address these issues.

**Figure 1:**
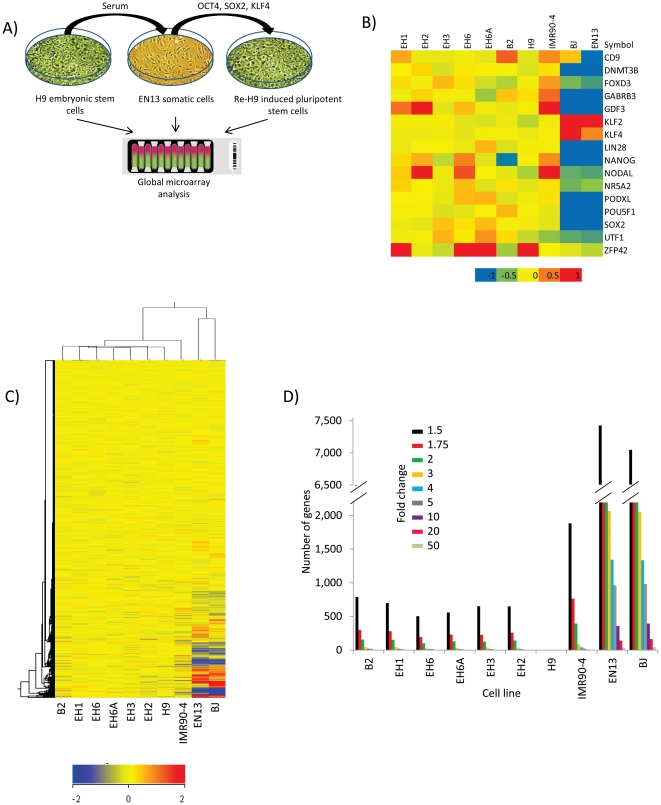
Derivation of re-H9 hiPSCs and their global transcriptional analysis. (**A**) Schematic of derivation of EN13 and re-H9 hiPS cell lines. (**B**) A transcriptome heatmap putative pluripotency markers. Quantile normalized, Log_2_ scale, and color range has been scaled to min/max of −1/1. (**C**) Global hierarchical clustering of gene expression microarray data for hESC (H9), hiPSCs (re-H9, IMR90-4), and somatic (BJ, EN13) cell lines. The clustering of transcripts is determined by metric euclidean (ordinary) distance using centroid linkage (ie. Distance = 
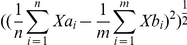
 where Xa, and Xb are the gene expression levels for two separate clusters, and n, m are the number of cluster entities, respectively). Groups which minimize the average distance between respective centroids are clustered together. Similar expression profiles are initially joined into groups. These groups are further joined in a dendrogram tree structure, and the process repeated until one single group contains all data. Thus, similarity increases for groupings with nodes towards the leaves (bottom of the tree). The scale used is log2, and the color has been scaled to a min/max of −2/2. (**D**) Graph of gene expression differences of hiPSC (re-H9, IMR90-4) and somatic cell (BJ, EN13) lines as compared to the hESC (H9) line. The number of genes is plotted on the vertical axis vs. cell line on the horizontal axis. The selected fold differences are between 1.5 and 50 with respect to hESC line H9.

In brief, the well characterized female hES cell line H9 was allowed to differentiate into a clonally purified mortal splanchnopleuric mesodermal somatic cell line EN13 [8]. The EN13 line was subsequently reprogrammed back to an induced pluripotent state (Fig. 1A) which we named re-H9 [4]. Our results reveal several important differences between embryo-derived H9 and the induced re-H9 stem cells. We find a dysregulation of genes involved in imprinting and altered expression of X-chromosome localized genes in re-H9 cells. Using high stringency we have identified neuronatin (*NNAT*) as the top candidate gene that shows suppression in our induced pluripotent re-H9 cell lines.

## Results and Discussion

### Hierarchical Clustering of Whole Genome Microarray Data

Characterization of EN13 was previously carried out by Biotime Inc. [8]. EN13 was derived through a large scale combinatorial method of progenitor cell derivation. This involved heterogeneous differentiation of H9 under multiple conditions, followed by subsequent culture in various stromal and epithelial cell media [8]. EN13 demonstrated normal telomere dynamics and proliferative capability as compared to neonatal foreskin fibroblasts.

Total RNA from six re-H9 clones was labelled and hybridized to an illumina HumanHT-12 v3 whole genome microarray. In the present study, the global transcriptomes of six independent re-H9 hiPSC clones, previously shown with immunohistochemistry, Q-PCR, and teratoma assay to be pluripotent [4], were compared to their genetically matched predecessor mortal cell line EN13 and the immortal hESC line H9. Gene array data for pluripotency markers (Fig. 1B) indicates that the re-H9 hiPSCs display similar levels to H9, while EN13 shows similar levels to the non-pluripotent line BJ. The genetically unmatched hiPSC line IMR90-4 derived from the female human diploid fibroblast line IMR90 and the human diploid fibroblast line BJ were used as comparison controls. Using this system of ten cell lines we were able to show that H9 hESCs and re-H9 hiPSCs are far more similar than anticipated (Fig. 1C, D) (as few as 9 genes deviate at a five fold difference).

Hierarchical clustering of all ten cell lines clearly revealed the striking similarity of the transcriptomes of isogenic re-H9 hiPSCs to H9 (Fig. 1B). Up to 99.6% of genes were expressed at similar levels between genetically matched hESCs and hiPSCs. The transcriptionally unrelated somatic cell lines BJ and EN13 grouped in a separate branch indicating that EN13 has a differentiated transcriptome and is highly dissimilar to hESC line H9 or hiPSCs (Fig. 1B, C, D). Furthermore, our isogenic hiPSC lines clustered with H9, indicating that they are more hESC-like. However, IMR90-4 hiPSCs were clustered in a separate branch from the hiPSC (re-H9s)/hESC H9 branch (Fig. 1C). This indicates that the re-H9 hiPSCs are transcriptionally more similar to the hESC line H9 than to the genetically unrelated hiPSC line IMR90-4. This is likely due to both differing genetic backgrounds and derivations conditions and therefore justifies the use of an isogenic system.

When we plotted the number of genes with differential folds from the hESC line H9, the results revealed a striking similarity of the isogenic re-H9 hiPSCs to H9 (Fig. 1D). When the re-H9 hiPSCs were compared to hESC line H9, there was a range of 102–154 genes (>2 fold), and 9–20 genes (>5 fold) differences. This is in stark contrast to the 1267 genes at five fold difference previously identified between non-isogenic hESCs and hiPSCs [1], validating the advantages of an isogenic approach. The unrelated IMR90-4 hiPSC line, on the other hand, has almost 3.9 times as many gene differences (>2 fold from H9) as compared to the isogenic re-H9 hiPSC line EH6 (Fig. 1D).

### Transcriptional Status of Imprinting in H9 re-iPSCs

**Figure 2:**
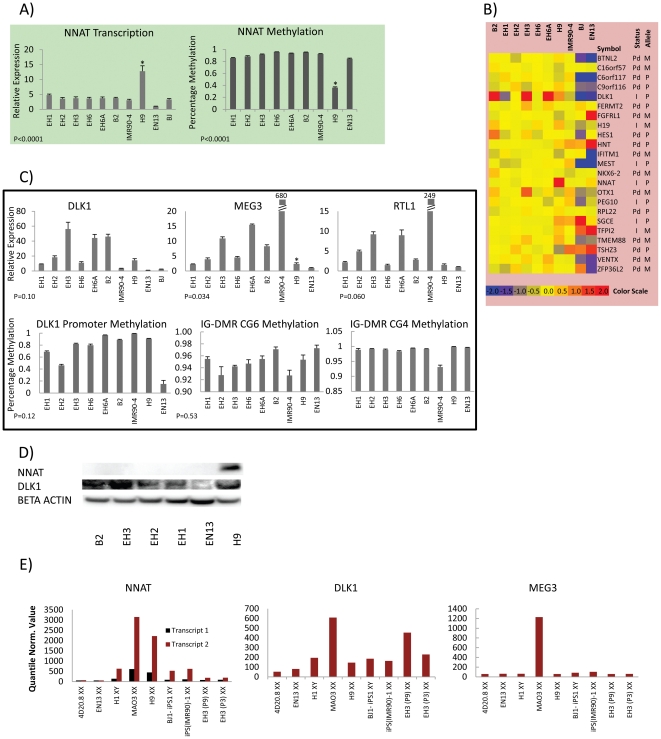
Altered imprinting profiles in hiPSCs. (**A**) **Left Plot:** Results of Q-PCR analysis of the imprinted neuronatin (*NNAT*) gene. **Right Plot:** Results of methylation specific Q-PCR for *NNAT* promoter. The error bars give the standard error. P-values are for α = 0.05, re-H9 hiPSCs vs. mean value of H9 hESC. * above H9 if P-value is significant. (**B**) A transcriptome heatmap of genes with putative imprinting functions that show at least a 1.5 fold difference from hESC line H9. Status: Pd = predicted to be imprinted, I = known to be imprinted. Expressed Allele: M = maternal, P = paternal. Log_2_ scale, and color has been scaled to min/max of −2/2. (**C**) **Top:** The Q-PCR results of the imprinted genes in the *DLK1-MEG3* region. **Bottom:** Results of methylation specific Q-PCR for *DLK1* promoter. The error bars give the standard error. P-values are for α = 0.05, re-H9 hiPSCs vs. mean value of H9 hESC. * above H9 if P-value is significant. (**D**) Western blot for protein expression of NNAT and DLK1. (**E**) Quantile normalized value of transcript expression for *NNAT* and *DLK1* with hESC lines H1, MAO3, H9, and hiPSC lines IMR90-1, BJ1-iPS1, EH3. Extracted from previously published microarray data collected by Biotime Inc. [4]. The sex of each cell line is indicated.

When we screened for genes with >10 fold difference between H9 and all re-H9 hiPSCs, only one gene was detected. The identified gene, neuronatin (*NNAT*), is a maternally silenced micro-imprinted gene [9] that is primarily expressed during early hindbrain development [10]. Compared to hESC line H9, the *NNAT* transcript was suppressed (by a mean of 3.5× by Q-PCR) in all the re-H9 hiPSCs (Fig. 2A), and Western Blotting confirmed similar results for NNAT protein (Fig. 2D). Additionally, while non-isogenic, and thus not as rigorous a test, *NNAT* transcript appeared to be at low levels in the genetically unrelated IMR90-4, BJ1, and IMR90-1 hiPSC lines as compared to female MAO3, and H9 hESC lines (Fig. 2A, E), but not significantly as compared to the male H1 hESC line. Methylation specific Q-PCR of the promoter region of *NNAT* correlated with epigenetic silencing of the transcript in the hiPSCs (Fig. 2A). Re-H9's compared as a group to the mean of H9 give a p-value<0.0001 for both gene expression and promoter methylation. These trends indicate that high levels of *NNAT* may be an important biomarker of a female hESC-like state.

This observation further prompted us to investigate other genes involved in the imprinting process. We identified several other alterations in a large group of imprinting genes. Transcriptional heatmaps of putative imprinting genes (>1.5 fold) in at least one re-H9 hiPSC line as compared to H9 are shown in Fig. 2B.

Imprinting errors in the *DLK1-DIO3* region (containing the gene *RTL1*, required for neonatal viability [11]) have recently been implicated in the embryonic non-viability of murine iPSCs during tetraploid complementation [12], [13], and also during somatic cell nuclear transfer in mice [14]. Our results indicated that the transcriptional levels of *DLK1/MEG3/RTL1* genes and DLK1 protein levels in hiPSCs were generally variable (Fig. 2B, C, D), with three out of six lines showing significant overexpression as compared to their parental line H9. Methylation specific Q-PCR (MS-QPCR) of the promoter region of *DLK1* along with CG4 and CG6 of the IG-DMR (as defined in [15], [16]) showed extensive methylation. The level of methylation when compared to murine studies [12], [17], [18] suggests that this may represent effective silencing of the region despite transcription that varies over two orders of magnitude between IMR90-4 and H9. However, co-expression of DLK1, and MEG3 (GTL2) in mice would normally occur in mice for hypomethylation of the maternal allele [17]. Neither methylation nor transcript levels showed significance when re-H9 hiPSCs were compared as a group to H9 hESC mean value in a t-test. This does imply that individual clones are not different, and over-expression of *RTL1* has been shown to cause imprinting defects leading to foetal lethality in mice [11]. Genetically unrelated hESC line H1 and hiPSC lines IMR90-1 and BJ1-IPS1 showed similar expression to H9 (Fig. 2E). However, hESC line MAO3 exhibited much higher expression of both DLK1 and MEG3 than either H1 or H9, and all unrelated lines co-expressed both DLK1 and MEG3. Thus, it is unclear what the “normal” state of the inner cell mass of human blastocysts should be, and the incredible variability between hESC lines warrants further investigation. Because of the variability, it is difficult to say what level can be considered silenced, and what proper methylation should be. Additionally, bisulfite conversion based measurements are indifferent to hydroxymethylation, which does not typically correspond with expression and may be an additional confounding factor [19]. Alterations in methylation and gene expression have also been observed to occur as a result of prolonged passaging in-vitro [20], [21], [22]. Despite these possibilities, we can assert that re-H9 hiPSCs show strong similarity, in transcriptional expression and in DNA methylation, to H9 hESCs. If H9 is “normal” the re-H9's are not aberrant.

**Figure 3:**
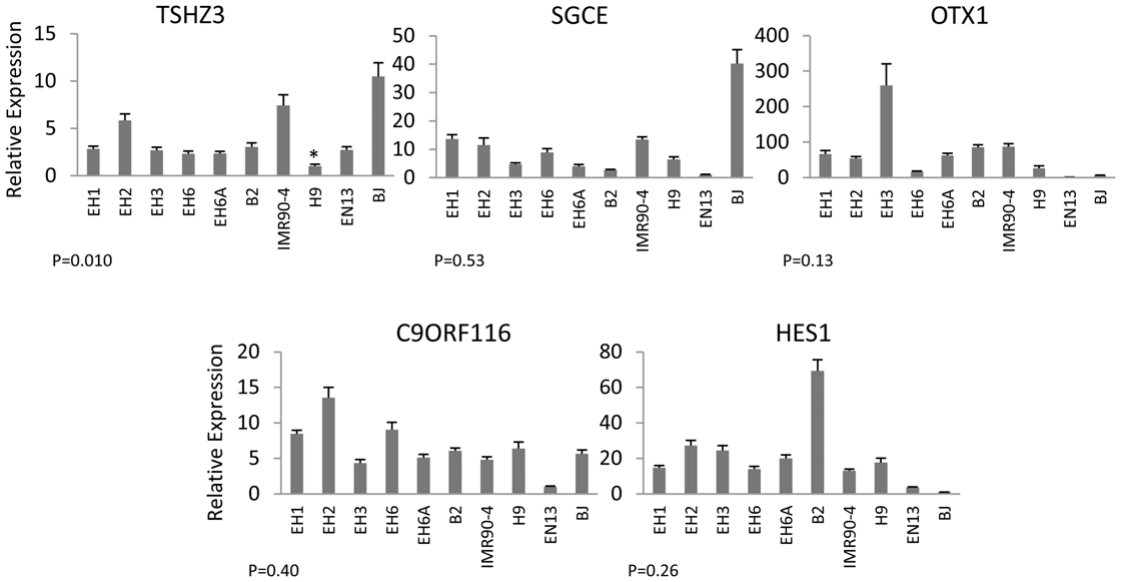
Q-PCR results for other regions with putative imprinting functions. Q-PCR analysis graphs indicate the relative expression of RNA in hESC (H9), hiPSCs (re-H9, IMR90-4) and somatic cell (BJ, EN13) lines for putatively imprinted genes with identified fold changes during transcriptional analysis. The error bars give the standard error of measurement. P-values are for α = 0.05, re-H9 hiPSCs vs. mean value of H9 hESC. * above H9 if P-value is significant.

Nevertheless, our results support a model in which imprinting defects are produced during iPS cell generation [12]. When q-PCR was performed on putatively imprinted genes displaying (>1.75 fold) differences on the microarray, some showed more significant differences (2× greater fold) (Fig. 3). Other putatively imprinted genes measured by q-PCR (Fig. 3) tend to show differences in only selected cell lines, with the exception of *TSHZ3*, and *OTX1* which were consistently over-expressed in most of the re-H9 hiPSC lines. Collectively our results show that a large intra-clonal variation in expression of imprinting genes exists, strongly suggesting that induced reprogramming can contribute to dysregulation of genes involved in genomic imprinting.

### Transcriptional Detection of Partial X-Chromosome Reactivation

**Figure 4:**
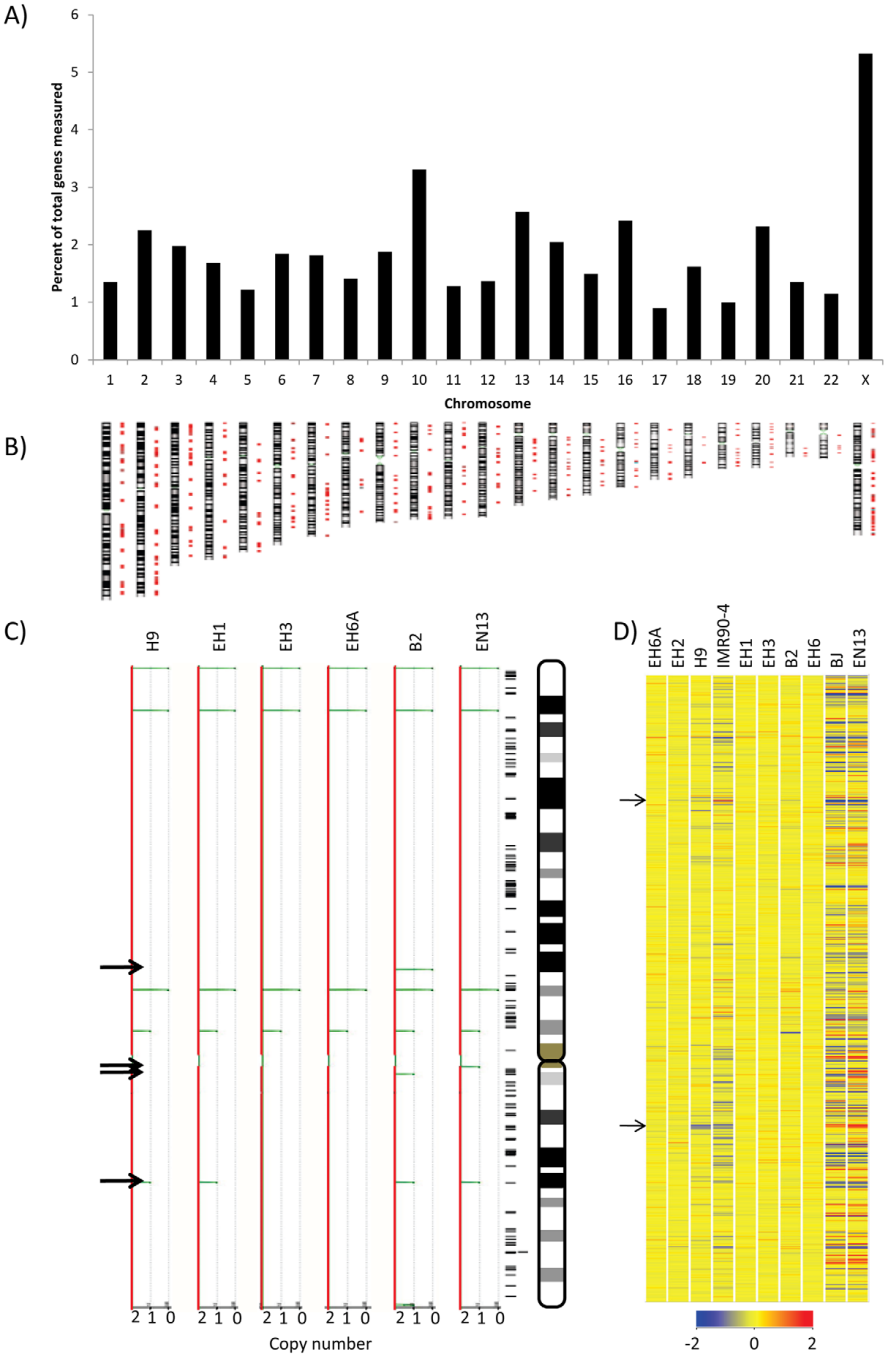
X-chromosome analysis of hiPSCs and H9. (**A**) Graphical representation of the number of genes with two fold or greater differences in at least one re-H9 hiPSC line as compared to the hESC line H9. The percentage of measured genes with two fold differences on a given chromosome is plotted on the vertical axis vs. chromosome number on the horizontal axis. (**B**) Gene density maps of loci from Fig. 3A, exhibiting at least two fold differences from hESC line H9, and their approximate chromosomal locations. (**C**) Copy number variations for the X-chromosome from Infinium HD high resolution (median spacing 1.2 Kb). SNP microarray data plotted for hESC line H9, and randomly selected re-H9 hiPSC lines (EH1, B2, EH3, EH6A) and the somatic cell line EN13. All variations observed on the X-chromosome are deletions. Deletions are indicated by green deviations from the red HapMap baseline (copy number 2). The hESC line H9 CNVs represent our normal karyotype. Arrows indicate areas with possible de novo CNV's (deviations from normal karyotype hESC line H9). Loci of genes with 1.5 fold difference are plotted on the right for comparison, with chromosome orientation indicated. (**B**) A heatmap of the transcriptional profile of the X-chromosome, sorted by gene locus. Several regions of incorrectly reprogrammed genes can be visualized as a shift toward blue color as compared to hESC line H9. The scale used is Log_2_ and the color has been scaled to a min/max of −2/2.

Next we wanted to determine if the transcriptional changes we observed have chromosome-specific associations. We selected genes that were expressed differentially between at least one re-H9 hiPSC and H9 (>2 fold) and plotted the percentage of affected genes (either up or down regulated) vs. chromosome number (Fig. 4A). This analysis revealed a significant anomaly on the X-chromosome. About 5.3% of the X-chromosome genes were differentially expressed in at least one hiPSC as compared to an average of 1.9% for the autosomes.

If, on the whole, partial reprogramming is a stochastic process, then one would predict a constant distribution of altered gene expression across chromosomes (1.9%). This is indeed correct for most autosomes (Fig. 4A, B). However, our results indicate twice as many altered genes on the X-chromosome (up or down regulated) (Fig. 4A). If H9 has an XaXi state (one active and one inactive X-chromosome), and the hiPSCs have an XaXa state, then we could have double the gene dosage and would expect two-fold up-regulation in all genes (100%). When we sorted the X-chromosome gene expression profiles (up or down >2 fold) and compared them to H9, we found that over 70% of altered X-chromosome-localized genes (3.8% of measured X-chromosome genes) were due to over-expression (Fig. S1). However, fewer than 100% of X-chromosome genes are expected to be up-regulated since up to 25% of the genes on the inactive X-chromosome may not be originally silenced [23], and gene dosage may not be at the same level when Xi is reactivated. The X-chromosome inactivation/re-activation has been seen to be dependent on *OCT4*, *SOX2* in mouse [24], [25], [26] and *OCT4*, *KLF4* in human [27], which we have used to reprogram EN13 somatic cells to re-H9 hiPSC. Consequently, we postulated that our results can be explained by partial de-repression of the Xi chromosome.

The changes on the X-chromosome genes we have observed could be directly due to alterations in the genome. However, when we performed high density SNP chip karyotyping on several of our hiPSCs (1.2 kb resolution) we observed no significant changes (Fig. 4C). Although minor alterations were observed (arrows), they were not localized to gene-rich areas or the position of the identified genes (Fig. 4C). Common Genomic Variant Region Analysis was performed for re-H9 lines, identifying aberrant regions in or near to genes. These aberrances were generally present in H9 itself, however, and thus represented a normal karyotype. Additionally, most other aberrations were inconsistently represented among the re-H9 groups (Fig. S2, S3, S4, S5, S6, S7, S8, S9, S10, S11, S12, S13), precluding the possibility that they were the cause of consistent trends such as *NNAT* silencing, or X-chromosome reactivation.

**Figure 5:**
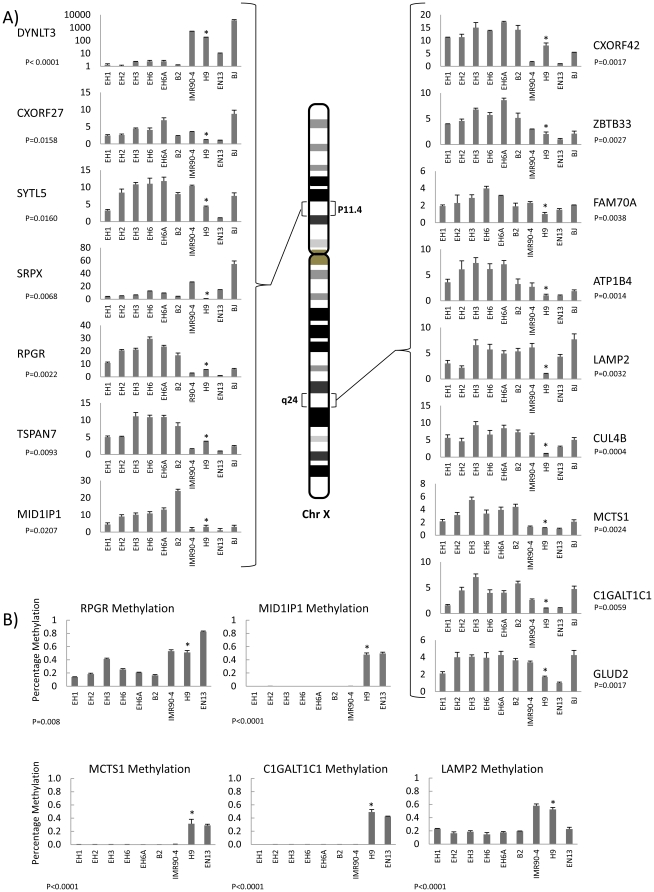
Q-PCR and methylation of X-chromosome dysregulated gene loci p11.4 and q24. (**A**) Identified gene loci that are up-regulated compared to hESC line H9, on the X-chromosome, sorted according to their location. Q-PCR graphs of relative expressions in H9 vs. hESC (H9), hiPSCs (re-H9, IMR90-4), and somatic cell lines (BJ, EN13) for selected genes from two regions on the X-chromosome. The investigated regions p11.4, and q24 are indicated, and genes are in the order that they would be found on the chromosome. P-values are for α = 0.05, re-H9 hiPSCs vs. mean value of H9 hESC. * above H9 if P-value is significant. (**B**) Methylation specific Q-PCR for the genes RPGR, MID1IP1, MCTS1, C1GALT1C1 and LAMP2 promoters are presented below the Q-PCR results of their respective regions. P-values are for α = 0.05, re-H9 hiPSCs vs. mean value of H9 hESC. * above H9 if P-value is significant.

When all X-chromosome localized genes were sorted according to their corresponding chromosome location (Fig. 4D), several hotspots of de-repression were observed (shown by arrows) including Xp11.4 and Xq24. The genes corresponding to some of these hotspots were identified, and verified with q-PCR analysis and sorted according to chromosome loci (Fig. 5). With the exception of *DYNLT3* transcript, which was consistently silenced, all other genes displayed were over-expressed. Therefore, we identified *DYNLT3* as another plausible biomarker of correct reprogramming.

To confirm that this transcriptional activation is indeed due to Xi de-repression, and not due to a doubling of transcription from the Xa chromosome we performed methylation specific Q-PCR for selected genes within the area of interest (Fig. 5B). Our results indicated that H9 had a methylated/unmethylated ratio of approximately 50% suggesting a state in which the gene is active on one x-chromosome, and inactive on the other, while the re-H9 hiPSCs exhibited significantly decreased methylation indicating partial or full biallelic expression. This implies a transition from an almost pure XaXi state in H9 to a XaXi' state in the re-H9 lines, where Xi' denotes partial activation.

It has been previously reported [28] that female iPSCs maintain an inactive X-chromosome using XIST staining to measure the activation status. However, we believe our results are consistent with the aforementioned measurements. XIST staining is likely only applicable to the bulk activation/deactivation of an X chromosome, as localized areas of transcriptional de-repression in Xi are not optically visible. X-chromosome inactivation in lieu of XIST staining has been previously observed in hESCs [29], [30], and hiPSCs [28]. It is possible that this results from extended passaging in non-ideal in-vitro conditions, and clonal selection pressure [31]. A recent comparison of female hiPSCs and hESCs is also in agreement with our results, suggesting both full and partial activation of the inactive X [32]. In addition, it is known that not all genes on Xi are inactivated, and that some genes may be heterogeneously inactivated in the human population [33]. The fact that all re-H9 hiPSCs share similar activation features is likely a consequence of similar derivation conditions, and similar genetic background. Our isogenic model may aid in teasing out the various contributions.

In this first isogenic transcriptome profile analysis of human iPSCs, we have shown that hiPSCs are far more transcriptionally similar to hESCs than previously anticipated. Even with this high similarity, we have found significant differences in several imprinting genes. Of particular note is the consistently silenced gene *NNAT*, which may prove useful as a biomarker of successful reprogramming. We also demonstrate that transcriptional profiling can be used to successfully identify alterations in X-chromosome activation that can also be used as a biomarker of evaluating female human induced pluripotent stem cells. Imprinting errors as a consequence of partial reprogramming by somatic cell nuclear transfer (SCNT) have been implicated in anomalous development of a number of animals [14], [21], [34], [35]. At least three quarters of natural fertilizations are non-viable as early as the implantation stage [36], and the SCNT procedure is even more error prone [37]. Our results and the aforementioned effects imply that generation of a “perfectly” reprogrammed cell is a rare event. Our results suggest that despite a 99.6% transcriptome similarity between hESCs and hiPSCs, the remaining 0.4% difference may contribute to significant alterations. Although it is currently unknown if imprinting and/or X-chromosome (in)activation errors lead to defective hiPSC-derived somatic cells for regenerative cell replacement functions, our study encourages further investigation into their normalcy and the necessity to carefully screen hiPSCs. Isogenic studies will be invaluable in deconvoluting the genetic differences between hESCs and hiPSCs and may lead to advancements in reprogramming efficiency and their safe use for regenerative medicine purposes.

## Materials and Methods

### Production of re-H9 IPS Cells and Cell Culture

The protocol has been detailed previously [4]. Briefly, EN13, a clonally purified mortal splanchnopleuric mesodermal somatic cell line derivative from H9 hESC line, was infected with SOX2, OCT4, and KLF4 using pMx retroviral vectors with 8 µg/ml polybrene. After 20 h they cells were plated on irradiated feeder cells, and switched to daily feedings of DMEM hESC media (Invitrogen; cat# 10829–018) with 16% KOSR media (Invitrogen). iPSC colonies were transferred to multi-well dishes on irradiated feeders for population expansion with daily hESC media changes. Following this, feeder-free 10 cm dishes were started on Matrigel (BD Bioscience) with mTeSR1 media (Stem Cell Technologies), supplemented with 100 ng/ml of basic FGF (Millipore). Colonies were fed daily, and differentiated colonies removed.

### Transcriptome Profiling

RNA was extracted using TRIzol and immediately frozen until further use. Once all RNA was collected, an RNeasy Mini Kit (Qiagen) was used to purify RNA from all samples at once using the standard protocol with standard modifications for purification of total RNA, including small RNA, and for TRIzol suspensions. On column DNase treatment was performed. RNA was standardized to 100 ng/µl and frozen for use with Illumina arrays.

RNA integrity was measured on an Agilent 2100 Bioanalyzer using RNA 6000 LabChips. All samples had RIN numbers of 10. cRNA was prepared using Ambion - Illumina TotalPrep-96 RNA Amplification Kit (cat# 4393543), according to the manufacturers protocol. Illumina HumanHT-12 V3 Gene Expressions chips were processed (hybridized, washed, stained) according to Illumina's standard protocols (Whole-Genome Gene Expression Direct Hybridization Assay Guide Part # 11286331 Rev. A) and scanned with an Illumina BeadArray Reader. Data extraction was performed using Illumina's BeadStudio Software.

Genomic DNA was collected by lysing hES or iPS cells in situ on Matrigel with SDS buffer containing proteinase K (Roche). The lysate was scraped, and DNA extracted using the standard phenol-chloroform method. DNA was checked for integrity on 1% agarose gels. Genotyping was performed with the Illumina Infinium HumanOmni1 Quad v1.0 Beadarray. Standard protocols (Infinium® HD Assay Super Protocol Guide Part # 11322427 Rev. B) were followed (whole genome amplification, fragmentation, precipitation and re-suspension of DNA, hybridization to beadchip, washing, extension, and staining) and scanned on an Illumina iScan system. SNP genotype calls were made using the default parameters in Genome Studio and CNV calls were made using the default parameters of the CNV partition plugin in Genome Studio. Data was exported for use in Genespring 11.0.2. All samples had SNP call rates of ≈99.7%. SNP figures were exported from tracks in Genesprings Genome Browser using homo sapiens Build hg19.

Gene expression data was analyzed in Genespring 11.0.2. Data was exported from BeadStudio into a compatible Illumina format. Data imported into Genespring was quantile normalized with median baseline subtraction. Data was analyzed on log2 scale. Fold differences were analyzed for comparison to the H9 cell line. Hierarchical clustering was carried out using a Euclidian distance metric with centroid linkage. Gene lists for the x-chromosome were exported from Genome Browser homo sapiens Build hg19. Gene lists for imprinted genes were obtained from http://igc.otago.ac.nz/home.html, and http://www.geneimprint.com. Lists were cross-referenced with NCBI for standard gene symbols, and imported into Genespring for further analysis.

Graphs of number of number of genes vs. cell lines, and number of genes vs. chromosome number, as well as some gene averaged heatmaps were produced in excel using automated macros. Lists were filtered for unique gene symbols (∼25000) to avoid inclusion of multiple transcript variants and erroneous genes.

All raw and processed data for gene expression microarrays and SNP karyotyping microarrays are deposited in NCBI's Gene Expression Omnibus (GEO) accessible through GEO series accession number GSE31845 (http://www.ncbi.nlm.nih.gov/geo/query/acc.cgi?acc=GSE31845).

### Quantitative RT-PCR

Primers were designed using NCBI's Primer-BLAST. Reactions were run in 384 well plates on a ABI 7900HT Fast Real-Time PCR System. RNA was quantified on a NanoDrop spectrophotometer (Thermo Scientific). Briefly, reverse transcription was carried out using a First-Strand cDNA Synthesis Kit (G.E. Healthcare). Each of the PCR-plate wells contained 5 µl of either Power SYBR Green PCR Master Mix (Applied Biosystems) or PerfeCTa SYBR Green SuperMix (Quanta Biosciences) was mixed with 1 µl of 10 mM primer in DEPC treated H2O and 2–4 µl of cDNA, depending on the primer. Cycling conditions consisted generally of 10 minutes at 95°C followed by 40cycles of 15 s at 95°C and 1.25 minutes at 59°C. Samples were run in triplicate with standards run in duplicate. Relative expression was calculated using the ΔΔCt method, normalized to GAPDH. Standard errors were calculated from triplicate technical replicate measurements.

### Methylation Specific Quantitative RT-PCR

Primers were designed using MethPrimer [38] after identification of promoter regions using the Transcriptional Regulatory Element Database (http://rulai.cshl.edu/cgi-bin/TRED/tred.cgi?process=home). Bisulfite conversion of phenol and silica column purified DNA was carried out according to standard protocol using a Qiagen EpiTect Bisulfite Kit. Following conversion Q-PCR was carried out in a 384 well plates on a Bio-Rad CFX384 Real-Time PCR Detection System. Reactions were carried out in 10 µl reactions with Power SYBR Green PCR Master Mix (Applied Biosystems). The amount of methylation was calculated using ΔCt method according to M/(M+U) where M represents the amount of methylated DNA and U the amount of unmethylated DNA. Standard errors were calculated from triplicate technical replicate measurements.

### Western Blots

Western blotting was carried out using NuPage 4–12% Bis-Tris gels with MES running buffer and TRIS-Glycine transfer buffer with 30% methanol. Cell lines for protein extraction were provided by Biotime Inc., and lysed in RIPA buffer. Antibodies were purchased from Santa Cruz: Neuronatin S-14 catalog #: sc-23437, and DLK1 H-118 catalog #: sc-25437.

### Statistical Analysis

Independent two tailed one sample t-tests were conducted for Q-PCR runs treating re-H9 hiPSCs as one group and comparing to the H9 hESC line mean for a given gene. Alpha was set at 0.05 and P-values are recorded in the lower left of graphs. These values do not indicate that any individual line is significant as compared to H9.

## Supporting Information

Figure S1 **Graphical representation of the number of genes with two fold or greater differences in at least one re-H9 hiPSC line as compared to the hESC line H9.** Yellow - the percentage of measured genes that are down-regulated (in all re-H9) with respect to H9. Black – the percentage of measured genes that are up-regulated (in all re-H9) with respect to H9.
(TIF)

Figure S2 **Chromosome 1 and 2 SNP chip copy number and log R ratios for hiPSC lines EH1, EH3, B2, EH6A, somatic cell line EN13, and hESC line H9.** Points are displayed non-averaged, and thus width of copy number variation is pixel limited, and does not represent true width.
(TIF)

Figure S3 **Chromosome 3 and 4 SNP chip copy number and log R ratios for hiPSC lines EH1, EH3, B2, EH6A, somatic cell line EN13, and hESC line H9.** Points are displayed non-averaged, and thus width of copy number variation is pixel limited, and does not represent true width.
(TIF)

Figure S4 **Chromosome 5 and 6 SNP chip copy number and log R ratios for hiPSC lines EH1, EH3, B2, EH6A, somatic cell line EN13, and hESC line H9.** Points are displayed non-averaged, and thus width of copy number variation is pixel limited, and does not represent true width.
(TIF)

Figure S5 **Chromosome 7 and 8 SNP chip copy number and log R ratios for hiPSC lines EH1, EH3, B2, EH6A, somatic cell line EN13, and hESC line H9.** Points are displayed non-averaged, and thus width of copy number variation is pixel limited, and does not represent true width.
(TIF)

Figure S6 **Chromosome 9 and 10 SNP chip copy number and log R ratios for hiPSC lines EH1, EH3, B2, EH6A, somatic cell line EN13, and hESC line H9.** Points are displayed non-averaged, and thus width of copy number variation is pixel limited, and does not represent true width.
(TIF)

Figure S7 **Chromosome 11 and 12 SNP chip copy number and log R ratios for hiPSC lines EH1, EH3, B2, EH6A, somatic cell line EN13, and hESC line H9.** Points are displayed non-averaged, and thus width of copy number variation is pixel limited, and does not represent true width.
(TIF)

Figure S8 **Chromosome 13 and 14 SNP chip copy number and log R ratios for hiPSC lines EH1, EH3, B2, EH6A, somatic cell line EN13, and hESC line H9.** Points are displayed non-averaged, and thus width of copy number variation is pixel limited, and does not represent true width.
(TIF)

Figure S9 **Chromosome 15 and 16 SNP chip copy number and log R ratios for hiPSC lines EH1, EH3, B2, EH6A, somatic cell line EN13, and hESC line H9.** Points are displayed non-averaged, and thus width of copy number variation is pixel limited, and does not represent true width.
(TIF)

Figure S10 **Chromosome 17 and 18 SNP chip copy number and log R ratios for hiPSC lines EH1, EH3, B2, EH6A, somatic cell line EN13, and hESC line H9.** Points are displayed non-averaged, and thus width of copy number variation is pixel limited, and does not represent true width.
(TIF)

Figure S11 **Chromosome 19 and 20 SNP chip copy number and log R ratios for hiPSC lines EH1, EH3, B2, EH6A, somatic cell line EN13, and hESC line H9.** Points are displayed non-averaged, and thus width of copy number variation is pixel limited, and does not represent true width.
(TIF)

Figure S12 **Chromosome 21 and 22 SNP chip copy number and log R ratios for hiPSC lines EH1, EH3, B2, EH6A, somatic cell line EN13, and hESC line H9.** Points are displayed non-averaged, and thus width of copy number variation is pixel limited, and does not represent true width.
(TIF)

Figure S13 **X Chromosome SNP chip copy number and log R ratios for hiPSC lines EH1, EH3, B2, EH6A, somatic cell line EN13, and hESC line H9.** Points are displayed non-averaged, and thus width of copy number variation is pixel limited, and does not represent true width.
(TIF)

## References

[1] Takahashi K, Tanabe K, Ohnuki M, Narita M, Ichisaka T (2007). Induction of pluripotent stem cells from adult human fibroblasts by defined factors.. Cell.

[2] Yu J, Vodyanik MA, Smuga-Otto K, Antosiewicz-Bourget J, Frane JL (2007). Induced Pluripotent Stem Cell Lines Derived from Human Somatic Cells.. Science.

[3] Feng Q, Lu SJ, Klimanskaya I, Gomes I, Kim D (2010). Hemangioblastic derivatives from human induced pluripotent stem cells exhibit limited expansion and early senescence.. Stem Cells.

[4] Vaziri H, Chapman KB, Guigova A, Teichroeb J, Lacher MD (2010). Spontaneous reversal of the developmental aging of normal human cells following transcriptional reprogramming.. Regen Med.

[5] Hu B-Y, Weick JP, Yu J, Ma L-X, Zhang X-Q (2010). Neural differentiation of human induced pluripotent stem cells follows developmental principles but with variable potency.. Proceedings of the National Academy of Sciences.

[6] Chin MH, Mason MJ, Xie W, Volinia S, Singer M (2009). Induced pluripotent stem cells and embryonic stem cells are distinguished by gene expression signatures.. Cell Stem Cell.

[7] Marchetto MC, Yeo GW, Kainohana O, Marsala M, Gage FH (2009). Transcriptional signature and memory retention of human-induced pluripotent stem cells.. PLoS One.

[8] West MD, Sargent RG, Long J, Brown C, Chu JS (2008). The ACTCellerate initiative: large-scale combinatorial cloning of novel human embryonic stem cell derivatives.. Regen Med.

[9] Evans HK, Wylie AA, Murphy SK, Jirtle RL (2001). The neuronatin gene resides in a “micro-imprinted” domain on human chromosome 20q11.2.. Genomics.

[10] Wijnholds J, Chowdhury K, Wehr R, Gruss P (1995). Segment-specific expression of the neuronatin gene during early hindbrain development.. Dev Biol.

[11] Sekita Y, Wagatsuma H, Nakamura K, Ono R, Kagami M (2008). Role of retrotransposon-derived imprinted gene, Rtl1, in the feto-maternal interface of mouse placenta.. Nat Genet.

[12] Stadtfeld M, Apostolou E, Akutsu H, Fukuda A, Follett P (2010). Aberrant silencing of imprinted genes on chromosome 12qF1 in mouse induced pluripotent stem cells.. Nature.

[13] Liu L, Luo G-Z, Yang W, Zhao X, Zheng Q (2010). Activation of the imprinted Dlk1-Dio3 region correlates with pluripotency levels of mouse stem cells.. Journal of Biological Chemistry.

[14] Cui X-S, Zhang D-X, Ko Y-G, Kim N-H (2009). Aberrant epigenetic reprogramming of imprinted microRNA-127 and Rtl1 in cloned mouse embryos.. Biochemical and Biophysical Research Communications.

[15] Kagami M, Sekita Y, Nishimura G, Irie M, Kato F (2008). Deletions and epimutations affecting the human 14q32.2 imprinted region in individuals with paternal and maternal upd(14)-like phenotypes.. Nat Genet.

[16] Kagami M, O'Sullivan MJ, Green AJ, Watabe Y, Arisaka O (2010). The IG-DMR and the *MEG3*-DMR at Human Chromosome 14q32.2: Hierarchical Interaction and Distinct Functional Properties as Imprinting Control Centers.. PLoS Genetics.

[17] Rocha STd, Edwards CA, Ito M, Ogata T, Ferguson-Smith AC (2008). Genomic imprinting at the mammalian Dlk1-Dio3 domain.. Trends in Genetics.

[18] Sato S, Yoshida W, Soejima H, Nakabayashi K, Hata K (2011). Methylation dynamics of IG-DMR and Gtl2-DMR during murine embryonic and placental development..

[19] Jin S-G, Kadam S, Pfeifer GP (2010). Examination of the specificity of DNA methylation profiling techniques towards 5-methylcytosine and 5-hydroxymethylcytosine..

[20] Wright K, Brown L, Brown G, Casson P, Brown S (2011). Microarray assessment of methylation in individual mouse blastocyst stage embryos shows that in vitro culture may have widespread genomic effects.. Human Reproduction.

[21] Chang G, Liu S, Wang F, Zhang Y, Kou Z (2009). Differential methylation status of imprinted genes in nuclear transfer derived ES (NT-ES) cells.. Genomics.

[22] Meissner A, Mikkelsen TS, Gu H, Wernig M, Hanna J (2008). Genome-scale DNA methylation maps of pluripotent and differentiated cells.. Nature.

[23] Carrel L, Willard HF (2005). X-inactivation profile reveals extensive variability in X-linked gene expression in females.. Nature.

[24] Navarro P, Chambers I, Karwacki-Neisius V, Chureau C, Morey C (2008). Molecular coupling of Xist regulation and pluripotency.. Science.

[25] Navarro P, Avner P (2009). When X-inactivation meets pluripotency: An intimate rendezvous.. FEBS Letters.

[26] Guo G, Yang J, Nichols J, Hall JS, Eyres I (2009). Klf4 reverts developmentally programmed restriction of ground state pluripotency.. Development.

[27] Hanna J, Cheng AW, Saha K, Kim J, Lengner CJ (2010). Human embryonic stem cells with biological and epigenetic characteristics similar to those of mouse ESCs.. Proceedings of the National Academy of Sciences.

[28] Tchieu J, Kuoy E, Chin MH, Trinh H, Patterson M (2010). Female Human iPSCs Retain an Inactive X Chromosome.. Cell Stem Cell.

[29] Silva SS, Rowntree RK, Mekhoubad S, Lee JT (2008). X-chromosome inactivation and epigenetic fluidity in human embryonic stem cells.

[30] Lengner CJ, Gimelbrant AA, Erwin JA, Cheng AW, Guenther MG (2010). Derivation of Pre-X Inactivation Human Embryonic Stem Cells under Physiological Oxygen Concentrations.. Cell.

[31] Dvash T, Lavon N, Fan G (2010). Variations of X Chromosome Inactivation Occur in Early Passages of Female Human Embryonic Stem Cells.. PLoS ONE.

[32] Bruck T, Benvenisty N (2011). Meta-analysis of the heterogeneity of X chromosome inactivation in human pluripotent stem cells.. Stem Cell Research.

[33] Carrel L, Willard HF (1999). Heterogeneous gene expression from the inactive X chromosome: An X-linked gene that escapes X inactivation in some human cell lines but is inactivated in others.

[34] Young LE, Schnieke AE, McCreath KJ, Wieckowski S, Konfortova G (2003). Conservation of IGF2-H19 and IGF2R imprinting in sheep: effects of somatic cell nuclear transfer.. Mechanisms of Development.

[35] Han DW, Im YB, Do JT, Gupta MK, Uhm SJ (2008). Methylation status of putative differentially methylated regions of porcine *IGF2* and *H19*.. Molecular Reproduction and Development.

[36] Edwards RG (2001). IVF and the history of stem cells.. Nature.

[37] Simerly C, Navara C, Hwan Hyun S, Chun Lee B, Keun Kang S (2004). Embryogenesis and blastocyst development after somatic cell nuclear transfer in nonhuman primates: overcoming defects caused by meiotic spindle extraction.. Developmental Biology.

[38] Li L, Dahiya R (2002). MethPrimer: designing primers for methylation PCRs.. Bioinformatics.

