# Supplementary figures and images for: Suppression of the Imprinted Gene NNAT and X-Chromosome Gene Activation in Isogenic Human iPS Cells
Source: PLoS One. 2011 Oct 12;6(10):e23436. doi: 10.1371/journal.pone.0023436 (PMC3192059; doi:10.1371/journal.pone.0023436)

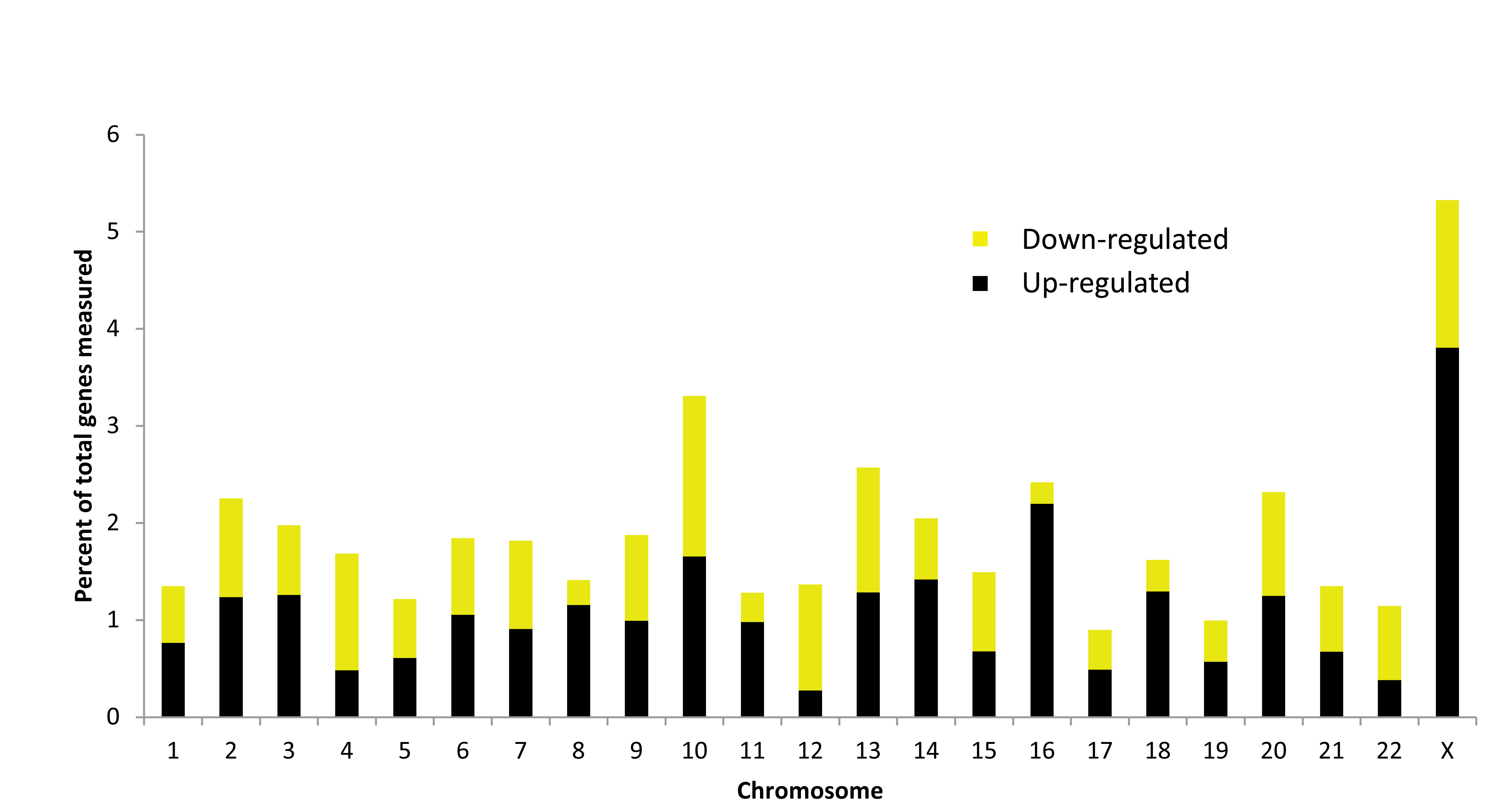

Supplement: Figure S1 — Graphical representation of the number of genes with two fold or greater differences in at least one re-H9 hiPSC line as compared to the hESC line H9. Yellow - the percentage of measured genes that are down-regulated (in all re-H9) with respect to H9. Black – the percentage of measured genes that are up-regulated (in all re-H9) with respect to H9. (TIF) [file pone.0023436.s001.tif]

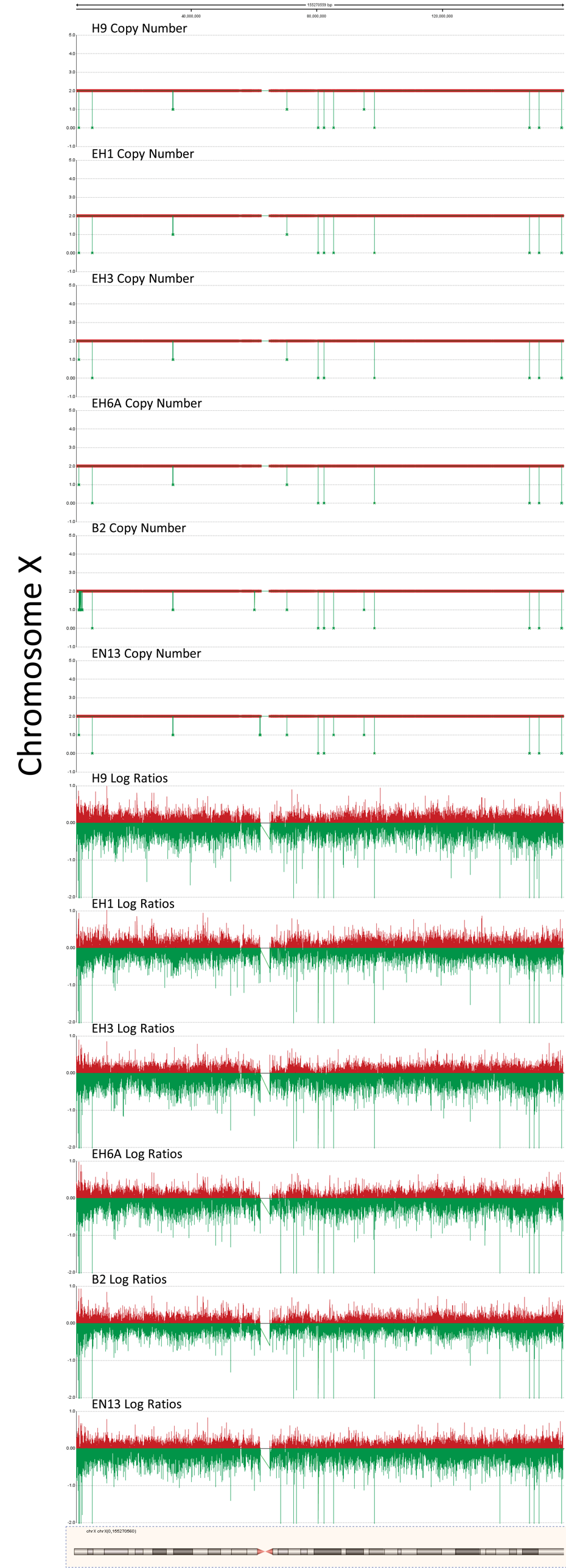

Supplement: Figure S13 — X Chromosome SNP chip copy number and log R ratios for hiPSC lines EH1, EH3, B2, EH6A, somatic cell line EN13, and hESC line H9. Points are displayed non-averaged, and thus width of copy number variation is pixel limited, and does not represent true width. (TIF) [file pone.0023436.s013.tif]
